# Not “out of Nantucket”: *Babesia microti* in southern New England comprises at least two major populations

**DOI:** 10.1186/s13071-014-0546-y

**Published:** 2014-12-10

**Authors:** Heidi K Goethert, Sam R Telford

**Affiliations:** Department of Infectious Disease and Global Health, Cummings School of Veterinary Medicine, Tufts University, 200 Westboro Rd, 01536 North Grafton, MA USA

**Keywords:** *Babesia microti*, New England, Epidemiology, VNTR, Emergence

## Abstract

**Background:**

Deer tick-transmitted human babesiosis due to *Babesia microti* appears to be expanding its distribution and prevalence in the northeastern United States. One hypothesis for this emergence is the introduction of parasites into new sites from areas of long-standing transmission, such as Nantucket Island, Massachusetts.

**Methods:**

We developed a typing system based on variable number tandem repeat loci that distinguished individual *B. microti* genotypes. We thereby analyzed the population structure of parasites from 11 sites, representing long-standing and newly emerging transmission in southern New England (northeastern United States), and compared their haplotypes and allele frequencies to determine the most probable number of *B. microti* populations represented by our enzootic collections. We expected to find evidence for a point source introduction across southern New England, with all parasites clearly derived from Nantucket, the site with the most intense longstanding transmission.

**Results:**

*B. microti* in southern New England comprises at least two major populations, arguing against a single source. The Nantucket group comprises Martha's Vineyard, Nantucket and nearby Cape Cod. The Connecticut/Rhode Island (CT/RI) group consists of all the samples from those states along with samples from emerging sites in Massachusetts.

**Conclusions:**

The expansion of *B. microti* in the southern New England mainland is not due to parasites from the nearby terminal moraine islands (Nantucket group), but rather from the CT/RI group. The development of new *B. microti* foci is likely due to a mix of local intensification of transmission within relict foci across southern New England as well as long distance introduction events.

**Electronic supplementary material:**

The online version of this article (doi:10.1186/s13071-014-0546-y) contains supplementary material, which is available to authorized users.

## Background

Babesiosis due to *Babesia microti* (Apicomplexa:Sporozoea) was first recognized as a zoonosis in 1969 when a resident of Nantucket Island, Massachusetts, sustained a malaria-like illness. Nearly a dozen cases were identified from Nantucket by 1976, leading to its colloquial name at the time, “Nantucket fever”. The white-footed mouse, *Peromyscus leucopus,* was incriminated as a main reservoir, with deer ticks (*Ixodes dammini*; also known as the northern population of *Ixodes scapularis*) as the vector [1,2]. Classic epidemiological and ecological studies by Spielman and Piesman described the enzootic cycle of *B. microti* on Nantucket and elsewhere in coastal New England, subsequently providing the basis for our rapid understanding of that of the spirochetal agent of Lyme disease (*Borrelia burgdorferi*), once *I. dammini* was incriminated as the vector of that bacterial zoonosis. Other animals (shrews, voles, chipmunks, squirrels) have been found infected by *B. microti* but their reservoir capacity remains largely undescribed [1,3]. The potential for enzootic transmission of *B. microti* would seem to rest mainly on the presence of a suitable *Ixodes* sp. vector and small rodents or insectivores.

For the first two decades after the index case of Nantucket fever, babesiosis was considered a rare infection that affected residents of the terminal moraine sites of New England and New York (Nantucket, Martha’s Vineyard, Cape Cod, Block Island, eastern Long Island), as well as a focus in the upper Midwest [4]. In addition, babesiosis was thought to affect only those people who were elderly, immune compromised or asplenic, thereby limiting its perceived public health significance despite the fact that those individuals sustained severe complications with a case fatality rate of 6- 9% [5]. In young, healthy individuals, infection with *B. microti* is usually asymptomatic and treatment may not be indicated. However, asymptomatic infection in healthy individuals threatens our blood supply, and more than 160 babesiosis cases have been acquired by transfusion. Many transfusion recipients are immunocompromised or have comorbidities and thus severe disease may result; about 20% of these cases are fatal. Babesiosis is now considered the greatest threat to our blood supply due to an infectious agent [6]. Accordingly, our perception of the clinical significance of babesiosis has changed over the last two decades.

Within the last decade, the northeastern U.S. has experienced a rising incidence of disease, although there is some confounding due to the fact that few states mandated reporting, and babesiosis only became nationally reportable in 2011. There has been a marked expansion of the geographic range of human disease. Cases have now been diagnosed in all New England states, north into Maine, west into the upper Hudson River valley, eastern Pennsylvania and New Jersey and as far south as Maryland [7-9]. This expansion has not been limited to the northeastern U.S. The Midwestern foci in Minnesota and Wisconsin has also intensified in transmission and expanded in distribution [10,11]. The biological basis for the expanded distribution remains undescribed. The public health burden of Lyme disease has changed significantly within the last 30 years, generally as a result of the expanded range and increased density of the tick vector, a function of dispersal by birds, habitat fragmentation, and burgeoning deer herds. However, the dramatic change in Lyme disease risk over time was not accompanied by that of babesiosis, at least until the last decade. For example, Westchester County, perhaps the epicenter of Lyme disease risk since the late 1980s, did not identify babesiosis in residents there until 2001 [12]. This lag in risk relative to Lyme disease was thought to be consistent with the mode of dispersal of the two infections, because *B. burgdorferi* could be transported by reservoir competent birds and subadult *I. dammini* infesting them; *B. microti* would inefficiently be introduced to new sites because birds are likely not reservoirs for this protozoan and transported nymphs would not seek competent hosts as adult ticks. The empiric evidence for a recently expanded geographic distribution of zoonotic *B. microti* is a paradox given these considerations. To determine whether the expanded distribution of zoonotic *B. microti* reflects the introduction of parasites from longstanding sites of transmission into new sites, we compared the genetic diversity of parasites from Nantucket and Martha’s Vineyard with that of those from mainland New England sites, including more recently established sites of transmission. In particular, we developed variable number tandem repeat (VNTR) markers based on the published *B. microti* genome [13], and used the resulting allele frequencies to determine the most probable number of *B. microti* populations represented by our enzootic collections. Limited genetic diversity across our sampling sites would provide evidence for a “transport-introduction” hypothesis to explain the recently expanded distribution of *B. microti* babesiosis.

## Methods

### Sources of *B. microti* DNA

Archived collections of tick or small mammal blood samples were mainly from long-standing field studies of *I. dammini* population biology [4,14,15] in coastal New England. Martha's Vineyard and Nantucket Island were the sites first experiencing zoonotic babesiosis [16]. *B. microti* was documented in rodents from Ipswich (Essex County), Sandy Neck and Great Island (Cape Cod, Barnstable County), Prudence Island and Block Island, Rhode Island; and coastal Connecticut by the 1980s [17-19]. In contrast, Dover (Norfolk County) and Grafton (Worcester County) in central Massachusetts are newly emergent transmission sites: the first cases of likely authochthonous babesiosis occurred in Norfolk County in the mid 2000s and, to date, there have been none documented in Grafton, even though there has been low level enzootic transmission there since at least 2002 (unpublished). Rodent blood was stored at -20C or dried on filter paper. Drag sampled host seeking nymphal deer ticks were stored desiccated, in 70% ethanol, or frozen until analysis; extracted DNA was also archived. DNA was extracted from blood using a DNEasy kit (Qiagen). Ticks were macerated individually and then extracted using a HOTSHOT protocol [20]. Extracted DNA was screened for the presence of *B. microti* DNA by PCR using the BmITS1F/BmITS1R primer set [21].

#### Identification and evaluation of tandem repeat markers

The *B. microti* genome (GenBank FO082871, FO08272 and FO082874) was searched for tandem repeats using the Tandem Repeats Finder (http://tandem.bu.edu/trf/trf.basic.submit.html). Primers targeting the flanking sequence were designed using Primer3 [22]. Amplification was done using Picomaxx (Agilent Technologies) high fidelity taq polymerase using a step-down cycling protocol; the concentration of each primer was 0.5 micromolar in each 15 uL reaction. Template concentration was not measured; 1.5 uL was added directly from the HOTSHOT extract. Amplification cycles were started at 65C, decreasing by 1 degree each cycle until 55C then 55 cycles were run at the 55C annealing temperature. The forward primers were fluorescently labeled using either FAM or HEX (Integrated DNA Technologies). Amplicons were then mixed with either the GeneScan500 (Applied Biosystems) or MapMarker1000 (BioVentures) ladders, depending on the expected size range, and sent to University of Maine Sequencing Facility at Orono, ME for accurate sizing on a capillary-based sequencer. The resulting data was analyzed by hand using PeakScanner (Applied Biosystems) and STRand software [23].

The amplicon from each potential tandem repeat locus was assessed for size differences between laboratory *B. microti* strains originating from Spooner, Wisconsin and Nantucket Island, Massachusetts, as well as the expected size from the genome posted on GenBank. If the marker was invariant among these samples, it was rejected. Markers were also rejected for failure to amplify well or for amplification of multiple bands. The final 9 loci were multiplexed for amplification as follows: BMV1 and 2, BMV 4 and 5, BMV 8 and 10, and BMV 13 and 23. BMV 20 was amplified alone. Samples that did not amplify well with the multiplex assays were repeated with each locus individually before being deemed a failure. The ability to detect multiple peaks was evaluated by mixing DNA from 2 samples with different genotypes together at varying concentrations. Minor peaks were counted if they were greater than 40% of the major peak. Samples that had more than 2 loci that failed to amplify to were excluded from further analysis. *Babesia microti* is haploid in the mammalian host but has diploid stages in its tick host [24]. As with recent malarial population structure studies ([25-27] among others), we simplified the genetic analysis by assuming that parasites are haploid. Samples that had multiple peaks were assumed to be due to infection with two separate parasites and were treated as such. Twenty five samples had 2 peaks in more than one locus. These were excluded from the analysis because we were unable to definitively identify the genotype of each haploid.

The fidelity of the markers was evaluated in 2 ways. In the laboratory, the well characterized GI (Harvard) strain [28] has been continuously cycled from hamster to subadult deer tick to hamster since its isolation from a Nantucket human case in 1981. Parasites at each stage (bloodstream infection, engorged larvae, molted infected nymphs) were typed to determine whether scoring the markers was sensitive to changes in ploidy over the transmission cycle. To determine the long term stability of the markers in the field, field samples (host-seeking ticks or rodent blood) collected on Nantucket Island from 1986 to 2013 were compared. Diversity indices and diversity permutation tests were calculated using PAST [29] and expressed with the 95% confidence interval around the index.

#### Identification of population clusters

The most probable number of populations (K) was estimated using the program Structure [30]. Structure uses a Bayesian approach in which it assigns individuals probabilistically to a predefined number of populations based on allele frequencies and without prior knowledge of geographic sampling. It is not straight forward to determine the optimal number of populations (K) for a given data set. We therefore calculated delta K, which is the second order rate of change in the likelihood of K. Simulation studies have shown that the modal value of this distribution represents the uppermost level of structure or the "true" K of a given set of data [31]. The height of the modal value indicates the strength of the signal detected by Structure. The probable evolutionary relationships (lineages) among our samples were analyzed using Phyloviz [32]. This program uses the eBURST algorithm to attempt to identify groups of related organisms and their founding genotype. It then creates a radial diagram predicting the patterns of descent to the other genotypes in the group.

In all years, mammals were collected under permits issued by the respective state divisions of fish and wildlife. Prior to 2003, when we were at Harvard University, mammalogy fieldwork did not require institutional animal care and use committee approval; all fieldwork since 2003 was conducted under research protocols approved by the Tufts University IACUC.

## Results

Nine VNTR loci were identified that could be reliably amplified and demonstrated to have size differences between the Massachusetts, Wisconsin and the sequenced genome (a Connecticut isolate). (Table 1) The attributes of the markers were examined using data from field collections of mice and ticks from Nantucket Island from 1987–2013. (Table 2) In general, the rate of failed amplifications was low (5% or less). However, BMV20 showed a markedly greater rate of failure (29%) compared to the other loci. Failure to amplify BMV20 in these samples was consistent even when we redesigned the primers to anneal to alternate nearby locations on the gene. BMV20 appeared to have a null mutant in these samples and was treated as a separate allele in the analyses.

**Table 1:** **The VNTR loci used in the study and the observed (or predicted for the genome) amplicon sizes from the initial screen for variability**

**Locus**	**Repeat length**	**Repeat motif**	**Primers (5' to 3')**	**Observed size (bp)**		
				**Genome**	**MA**	**WI**
BMV1	6	AGTTCT or AGGTCA	F- CAATCTATGAGGCATGCGATTC	346	340	340
			R- CTAAAAGGCCCGATGGTTCA			
BMV2	6	TATAAC	F-TCCAGTGACAATGACATATTTAAGCA	400	405	392
			R- TGTCCTCATTCTGAGCCACAGT			
BMV4	11	TTAGCTATGGG	F- ACCACCACCAGGCCTCTATG	405	361	295
			R- CTGGACCATGATTTGGTTGA			
BMV5	9	GCTGTATTT	F- AGGCCCCTGTTCATCACATG	415	317	317
			R- GGAATAGCCTCGAGTCCAGA			
BMV8	10	ACATACAGCG	F- AGGCCAGTGGAGCAGAGAAG	262	327	295
			R- CAAGCAATCGTCGCTGTATG			
BMV10	3	GAT	F- TTGTTGGTGTCCGGGTTGTA	303	305	298
			R- ATGCTATTGCCTCGCAACCT			
BMV13	15	TCCTTACTAGCCTTA	F- ACCGCTCCCGCACTTTAGTA	351	520	443
			R- CCTGCGGGTTCTACCACTCT			
BMV20	6	ATACTA	F- CAGGGTTTATGCGAAGAGTGG	713	713	754
			R- GTGCTGCAGGCTTCGATGTA			
BMV23	5	ATATA	F- CCGCCTCTCCTATTCCCCTA	322	275	270
			R- GAAGAACAGTTGGATGACTTCG			

MA Massachusetts, WI Wisconsin.

**Table 2:** **The diversity of the** ***B. microti*** **VNTRs on Nantucket Island, MA**

**Locus**	**Size range (bp)**	**No. alleles**	**Diversity**	**No. failed amplification (%)**
BMV1	340	1	nd	0
BMV2	405	1	nd	0
BMV4	308-473	16	0.69 [0.63, 0.75]	3 (2%)
BMV5	299-347	5	0.17 [0.11,0.26]	0
BMV8	171-245	3	0.03 [0.03,0.07]	0
BMV10	299-311	5	0.45 [0.37, 0.52]	0
BMV13	505-520	2	0.01 [0.01, 0.05]	7 (5%)
BMV20	647-725	9	0.28 [0.22, 0.41]	39 (29%)
BMV23	243-280	4	0.08 [0.04, 0.15]	7 (5%)

**Figure 1:**
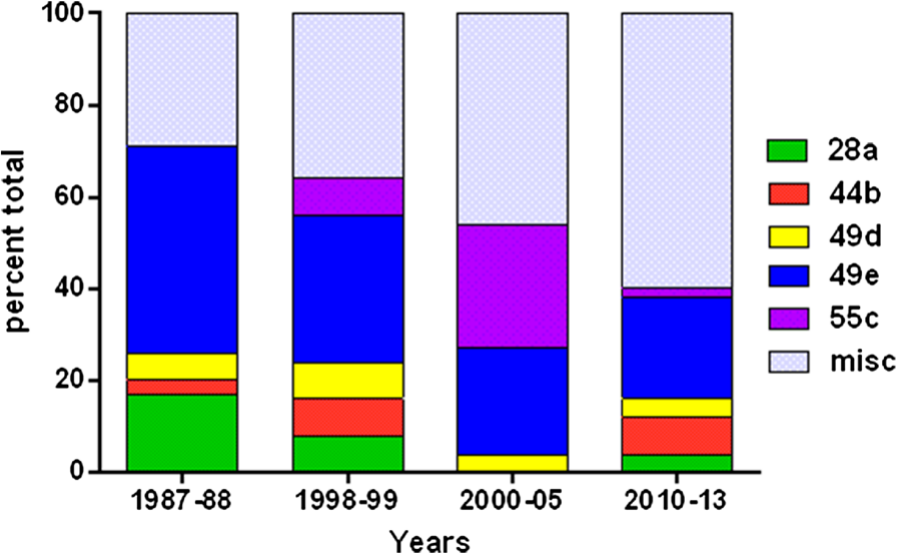
**The distribution of genotypes of** ***B. microti*** **collected on Nantucket by decade.** The contribution of the major genotypes is expressed as a percent of the total number of samples during that time.

The BMV loci are stable enough to provide consistency through a short transmission cycle, as amplicon size remained unchanged from all stages in the laboratory from the initial infection within a hamster to the larvae through the molt to nymphs and the resulting secondary infection (data not shown). Analysis of the Nantucket field samples showed that one genotype, 49e, remained dominant over a 26 year time span. (Figure 1) More than half (56%) of the samples collected had genotypes that were detectable over multiple decades. Three of the loci, BMV1, 2 and 13, were extremely stable and showed almost no variability over 26 years of sampling. The most diverse locus was BMV4 in which 16 different alleles were detected with a Simpson's index of diversity 0.69 [0.63, 0.75]. Interestingly, the diversity of parasites (and particularly the representation of miscellaneous haplotypes) appeared to increase over time.

**Figure 2:**
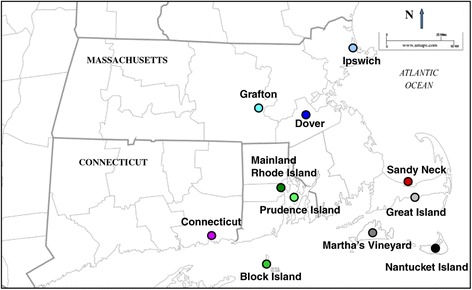
**Collection sites across southern New England.**

Samples were collected from 9 different sites across New England (Figure 2) representing sites with long-standing transmission, Martha's Vineyard, Nantucket Island, Ipswich, Great Island, Sandy Neck, Block Island, Prudence Island, mainland Rhode Island and Connecticut, and two with recent emergence of transmission, Dover and Grafton. From these, 387 samples were genotyped, and 190 unique types were obtained. (Table 3; Additional file 1) The Simpson’s diversity indices for the markers were large for every field site. They ranged from 0.51 [0.37, 0.63] on Great Island to 0.98 [0.97, 0.98] on nearby Martha's Vineyard.

**Table 3:** **Field sites sampled and the Simpson's index of diversity for identified genotypes**

	**Samples**	**Collection years**	**No. types**	**Diversity**	**No. with multiple types**
**Established sites of transmission**					
Martha's Vineyard	92	1999-2013	79	0.98 [0.97, 0.98]	19 (21%)
Nantucket	127	1986-2013	52	0.89 [0.85, 0.93]	30 (24%)
Great Island	58	1986, 1993	10	0.51 [0.37, 0.63]	17 (29%)
Sandy Neck	4	1988	4	nd	1 (25%)
Ipswich	10	1999, 2010	9	0.86 [0.79, 88]	5 (50%)
Block Island	11	1997-1998	8	0.80 [0.56, 0.85]	3 (27%)
Prudence Island	35	1998-2000	19	0.90 [0.84, 0.92]	8 (30%)
Connecticut	11	1997-2014	11	0.90 [0.78, 0.90]	2 (18%)
Mainland Rhode Island	4	2003	3	nd	0
**Emerging sites of transmission**					
Grafton	11	2008-2014	7	0.76 [0.51, 0.83]	5 (45%)
Dover	16	2012-2013	8	0.71 [0.53, 0.83]	2 (13)
**Total**	**387**		**190**	**0.97 [0.96, 0.98]**	

**Figure 3:**
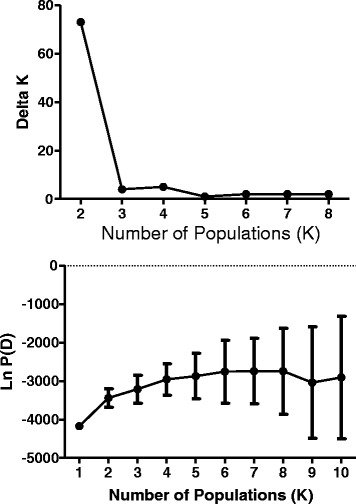
**The most probable number of populations (K) as calculated by Structure.** Bottom: The log probability for each number of populations (K); error bars represent the variance around each log probability, LnP(D). Top: Delta K of the number of populations (K); the modal value indicates the real K.

**Figure 4:**
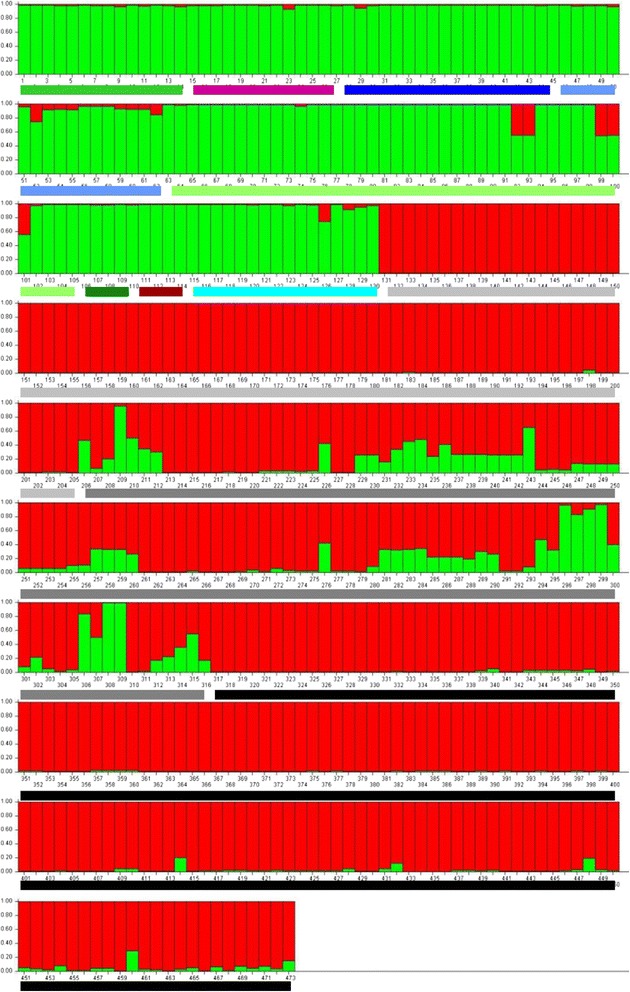
**The output from Structure for two populations (K = 2) demonstrating the placement of each sample (vertical bar) to either of the two main populations (Nantucket/MV, red; or CT/RI, green).** Horizontal lines under the X-axis designate the collection site of the sample; the color of these lines correspond to the color codes for each site as used in Figures 2 and 5.

The most probable number of populations (K) based on the sampled genetic variability was estimated using the program STRUCTURE. Delta (K) clearly demonstrates that the most likely number of populations for our New England data set is 2. (Figure 3) All Connecticut and Rhode Island samples were grouped together with the addition of mainland Massachusetts samples from Sandy Neck, Dover, Ipswich and Grafton. The second population consisted of samples from Nantucket, Great Island and most of the Martha’s Vineyard samples (Figure 4). Martha's Vineyard yielded samples from both populations; it may be that Martha’s Vineyard comprises the source for the two main *B. microti* populations demonstrated for southern New England, although we cannot exclude the possibility that the presence of the “mainland” population there represents a reverse introduction.

**Figure 5:**
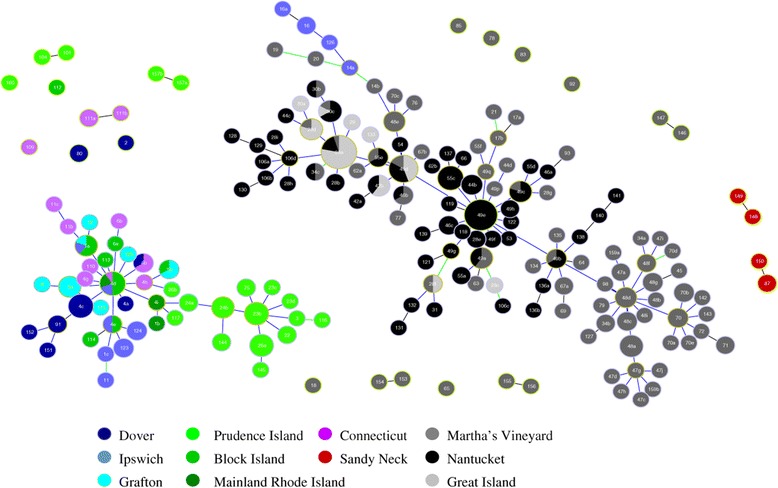
**Phyloviz analysis of** ***B. microti*** **samples from New England.** VNTR types were color-coded by geographic location. Colors correspond to those in Figures 2 and 4. Circle size is not directly proportional to prevalence. Lines imply ancestral-descendant lineage.

Phyloviz analysis also yielded two main clusters of samples (Figure 5), similar to those derived from the Structure analysis. Samples from Martha's Vineyard, Nantucket and Great Island comprise one cluster, while the samples from Connecticut, Rhode Island, Dover and Grafton, Massachusetts comprised the second. The samples from Sandy Neck did not cluster with either group. Samples from Ipswich were split between the two clusters. Although most of the samples from Martha's Vineyard were grouped together with the Nantucket and Great Island samples, a number of them fell all by themselves. None, however, clustered with the Connecticut and Rhode Island samples. Thus, an analysis that uses an algorithm to analyze lineages as opposed to allele frequencies would suggest the existence of more than 2 distinct *B. microti* populations, and that mainland sites of transmission do not derive from either Nantucket or Martha’s Vineyard, where human babesiosis due to *B. microti* was first recognized.

## Discussion

Our capacity to describe the population structure of *B. microti* has been hindered by the conserved nature of its genes. Sequencing of *B. microti* 18SrDNA, beta-tubulin, and ITS have demonstrated limited sequence diversity globally [33] and we previously were not able to reliably discriminate strains from Nantucket and Spooner, Wisconsin. With the availability of the *B. microti* genome [13], we developed an assay for typing *B. microti* based on variable number tandem repeats (VNTRs). Tandem repeat regions (or minisatellites) are portions of genes which rapidly evolve due to slip strand errors in replication, and have been used as the basis for typing diverse organisms, providing valuable insight into the ecology and epidemiology of the related *Babesia bovis* [34], *Theileria parva* [35], and *Plasmodium falciparum* [36,37]. Our VNTR markers appear to be ideal for molecular epidemiologic studies to analyze the zoonotic *B. microti* epidemic over time and space. Leveraging an extensive archive of Nantucket field samples we find that two markers, BMV1 and BMV2 did not change at all (no apparent production of new alleles) over 26 years. In contrast, BMV4 was highly variable yielding 16 different alleles (Simpson's index of diversity = 0.69). This balance of stability and variability in our VNTR marker set allows for measuring diversity without the loss of genotypic characterization due to homoplasy.

Since the recognition of *B. microti* babesiosis on Nantucket Island during the early 1970s and its subsequent characterization as “Nantucket fever”, this island has reported a large proportion of all nationally recognized cases [38,39]. The early force of *B. microti* transmission there stimulated the hypothesis that the guild of *I. dammini*-maintained microbes was originally to be found in the terminal moraine sites that were once contiguous with (given lower sea levels) or quickly reinvaded from southern refugia, likely Georges’ Island, after the retreat of the Laurentide ice sheet [40] about 6,000-12,000 years before present. Any newly recognized sites, particularly on mainland New England, were thought to represent introduction from original transmission sites such as Nantucket. Our analysis, however, clearly refutes anecdotal suggestions that Nantucket Island itself is the source of the recent geographic expansion of *B. microti* in New England. Parasites from the sites with emerging populations, Dover and Grafton, are more closely related to those from Connecticut and Rhode Island than those from Nantucket Island, a consistent finding by both methods of analysis (Structure and Phyloviz).

The haplotypes of *B. microti* collected across New England comprise at least two distinct populations. Parasites from the coastal islands of Massachusetts, Martha's Vineyard and Nantucket, along with samples from Great Island (25 km north of Nantucket, on Cape Cod) are grouped together (Nantucket group). Distinctly separate from these are all the parasites from Connecticut and Rhode Island, which includes samples from their coastal islands, Block Island and Prudence Island, (CT/RI group). The island of Martha's Vineyard yielded the most diverse population of parasites. Most of the samples clustered closely with those from Nantucket. However, Structure analysis groups some individual samples from Martha's Vineyard with CT/RI group, and for some samples no clear decision was made. Based on haplotypes, Phyloviz places these samples as singlets or doublets (unique haplotypes or those found only twice) with no clear connection to either main group. The presence of greater parasite diversity on Martha’s Vineyard may relate to the continuous survival of large tracts of oak woodland and scrubland for the past 1000 to 2000 years [41], whereas virtually all of Nantucket had been converted to sheep pasture by the mid 1800s. The deer tick was not present on either island during the first third of the 20^th^ century. An arthropod survey on Nantucket [42] lists only *Dermacentor variabilis* and the description of *I. muris* (Nantucket is the type locality) does not mention other ticks [43]. Intensive *D. variabilis* control programs on Martha’s Vineyard by Harvard’s Burt Wolbach during the late 1920s, continued by Marshall Hertig into the early 1940s [44], document the presence of “*I. scapularis*” on adjacent Naushon Island but not on the Vineyard, where *I. muris* was present. Indeed, in a seminal report describing “*Cytoecetes microti*”, now known as *Anaplasma phagocytophilum* [45,46] Tyzzer noted the presence of *Babesia* in splenectomized voles from Martha’s Vineyard, suggesting its enzootic transmission by *I. muris* [15]. Parasites maintained by the nidicolous tick, *I. muris,* would have been limited in their local distribution with few opportunities for recombination, due to the confinement of all 3 stages on rodents or within their nests. With the establishment of the 3-host tick *I. dammini*, presumably from nearby Naushon [47], small isolated natural foci could expand due to dispersal by the more diverse *I. dammini* hosts; such foci would then coalesce across the adaptive landscape of each island, as we have suggested for the agent of tularemia on Martha’s Vineyard [48]. Then too, the Nantucket landscape has changed even within the last 30 years, with greater portions of low-lying heathland overgrown by scrub oak thickets, thereby promoting denser populations of ticks and mice.

The history of zoonotic babesiosis due to *B. microti* is a function of the expanded distribution of dense infestations of *I. dammini* [15]. (It is noted that many consider *I. dammini* to simply be *I. scapularis* , but northern populations are genetically more homogenous than are those from more southerly sites [49-51] which represent ticks with limited anthropophily [52]). The stability and intensity of populations of deer ticks depend on that of deer [53]. Deer had largely been extirpated in New England by the late 1800s and forests were replaced by farmland [54] where the deer tick likely became very rare. Hence, early explanations of the origins of the Lyme disease and babesiosis epidemic emphasized the early report from Wolbach’s group [55] of the presence of “*I. scapularis*” on the Elizabeth Islands just north of Martha’s Vineyard because deer and forests had been protected there since the first days of colonization [56]; Naushon, the largest of the Elizabeth Islands, was thought to be the main postcolonial deer tick refugium until deer returned in numbers to other sites. Deer were, in fact, not completely extinct elsewhere in New England. Small remnant populations remained in numerous sites across New England, on Cape Cod, in the Berkshires, as well as sites in Connecticut and New York State [57]. These sites could have maintained unnoticed deer tick populations with their microbial guild; the absence of reports of collections of deer ticks from elsewhere does not imply evidence of absence in such sites. Isolated collections of *I. dammini* were in fact documented from Long Island, New Haven CT, and Cape Cod MA in the 1930s, and as far inland as Walpole, MA in 1949 [47]. Accordingly, the presence of a second major group of *B. microti* haplotypes (CT/RI) on the mainland should not be considered surprising. Infestations of deer ticks may have remained unrecognized until deer became locally abundant. Human babesiosis would emerge as the local force of transmission changed as a function of tick density, although increased suburbanization also was required to expose humans to infected ticks. A similar scenario of widely distributed relict populations across the northeastern U.S., with recent expansion from such cryptic sites as a result of greater deer tick density due to deer abundance and habitat change, has also been suggested for *B. burgdorferi* [58]. Inasmuch as our study was limited to convenience samples from southern New England, it is likely that additional distinct populations will be found across the range of enzootic *B. microti* in the Northeast. It would be particularly illuminating to analyze parasites from Shelter Island (eastern Long Island), where babesiosis cases were first identified at nearly the same time as those on Nantucket and Martha’s Vineyard.

Interestingly, parasites from Sandy Neck, a 7 km long x 1 km wide barrier beach 8 km due north of Great Island, were quite unique and did not consistently group with either population. Based on allele frequences, Structure placed Sandy Neck parasites with the CT/RI population (Figure 4). However, the haplotypes found there were unique and significantly different than those found elsewhere (Figure 5). Sandy Neck formed no more than 4,000 years ago [59] from accretion on the Barnstable moraine and comprises old stands of maritime pitch pine and oak forest. It was one of the early Massachusetts sites with a stable infestation of *I. dammini* and ecological studies of the tick began there in 1983. The unique *B. microti* alleles suggest isolation from Cape Cod (the barrier beach is connected at one side and physically separated for most of its length by a great salt marsh) and southern New England in general. However, we only analyzed a limited number of samples from that site and none that were recently acquired, and thus additional sampling may disprove the hypothesis of allopatry.

The fact that Ipswich yielded *B. microti* haplotypes from both populations is puzzling. The Ipswich area is adjacent to a major stopover for migratory birds (Parker River National Wildlife Refuge [60]) and indeed Ipswich was the northernmost zoonotic site (Lyme disease and babesiosis) in New England during the late 1980s [61], with a large distributional gap between that site and southern Massachusetts. Northward transport of infected nymphal ticks by migratory birds would be the most logical hypothesis to explain the development of foci north of the coastal infestations. However, if such ticks fed to repletion and developed to adults in the new site, they would not feed on reservoir competent hosts because adult deer ticks have never been found on rodents or insectivores. Although the reservoir competence of mustelids and carnivores (both possible hosts for adult deer ticks) for *B. microti* has not been formally explored, we have never detected natural infection in skunks, raccoons (these two hosts have their own seemingly specific *Babesia spp*. [33]), domestic dogs, and cats (unpublished) and they are unlikely candidates to initiate transmission in a new site. Deer are not susceptible to *B. microti* infection [62] and co-feeding transmission on such an incompetent host would be unlikely unless babesial sporozoites are pluripotent and could quickly develop to gametocytes. Cottontail rabbits are infected by *B. microti* [2,63] and uncommonly serve as host for adult *I. dammini* [63,64]. It is possible that they could serve as the “bridging” host for introduction. Alternatively, infected nymphal deer ticks may not feed to repletion on a bird, detach, and infest rodents to complete feeding, thereby introducing *B. microti* to competent reservoirs in new sites. It is also possible that infected rodents or even infected host-seeking ticks are inadvertently transported within or on luggage or household goods of vacationers, introducing infection to their yards. Finally, avian piroplasms have been described [65] and we cannot exclude the hypothesis that certain birds may indeed be reservoir competent for *B. microti* without a formal experimental test. Given that there are several possible means of introduction, it is likely that the currently expanding pattern of zoonotic *B. microti* risk in southern New England reflects both geological and demographic history as well as transport. Additional analyses are required, with more comprehensive sampling schemes, to fully describe the basis for the expanded distribution of human babesiosis due to *B. microti*.

## Conclusions

We demonstrate that enzootic *B. microti* in southern New England is comprised of two distinct populations. The Nantucket population is limited to Martha's Vineyard, Cape Cod and Nantucket Island in Massachusetts, and is not the source for newly emergent mainland sites of *B. microti* transmission. The CT/RI population is found in Connecticut and Rhode Island, including their coastal islands of Prudence and Block, as well as Ipswich, Massachusetts and newly emergent sites in mainland Massachusetts. Geology, postcolonial faunal and floral changes, and recent long distance transport events have all contributed to the current distribution of enzootic *B. microti*, but their relative contributions remain to be fully described.

## Additional file

Additional file 1:
**Allele sizes (in base pairs) for the 190 genotypes identified in this study.**
