# Supplementary material for: Not “out of Nantucket”: Babesia microti in southern New England comprises at least two major populations
Source: Parasit Vectors. 2014 Dec 10;7:546. doi: 10.1186/s13071-014-0546-y (PMC4272771; doi:10.1186/s13071-014-0546-y)
Supplement: Additional file 1: — Allele sizes (in base pairs) for the 190 genotypes identified in this study. [file 13071_2014_546_MOESM1_ESM.doc]

| **Haplotype** | **BMV1** | **BMV2** | **BMV5** | **BMV*** | **BMV10** | **BMV13** | **BMV23** | **BMV20** | **BMV4** |
| --- | --- | --- | --- | --- | --- | --- | --- | --- | --- |
| 1a | 340 | 405 | 317 | 241 | 302 | 396 | 243 | 695 | 418 |
| 1b | 340 | 405 | 317 | 241 | 302 | 396 | 243 | 695 | 451 |
| 1c | 340 | 405 | 317 | 241 | 302 | 396 | 243 | 695 | **385** |
| 2 | **340** | 405 | 317 | 241 | 299 | 396 | 243 | 999 | 407 |
| 3 | 340 | 405 | 317 | 241 | 299 | 671 | 243 | 695 | 484 |
| 4a | **340** | 405 | 317 | 241 | 305 | 396 | 243 | 695 | 374 |
| 4b | 340 | 405 | 317 | 241 | 305 | 396 | 243 | 695 | 440 |
| 4c | **340** | 405 | 317 | 241 | 305 | 396 | 243 | 695 | 407 |
| 4d | 340 | 405 | 317 | 241 | 305 | 396 | 243 | 695 | 418 |
| 4e | 340 | 405 | 317 | 241 | 305 | 396 | 243 | 695 | 385 |
| 4g | 340 | 405 | 317 | 241 | 305 | 396 | 243 | 695 | 539 |
| 4h | 340 | 405 | 317 | 241 | 305 | 396 | 243 | 695 | 429 |
| 4i | 340 | 405 | 317 | 241 | 305 | 396 | 243 | 695 | 451 |
| 5a | 340 | 405 | 317 | 241 | 305 | 396 | 238 | 695 | 418 |
| 5b | 340 | 405 | 317 | 241 | 305 | 396 | 238 | 695 | 429 |
| 6a | 340 | 405 | 317 | 241 | 305 | 396 | 243 | 999 | 418 |
| 6b | 340 | 405 | 317 | 241 | 305 | 396 | 243 | 999 | 495 |
| 8 | 340 | 405 | 317 | 241 | 305 | 396 | 238 | 999 | 418 |
| 11 | 340 | 405 | 317 | 241 | 302 | 396 | 243 | 999 | 385 |
| 11b | 340 | 405 | 317 | 241 | 302 | 396 | 243 | 999 | 418 |
| 11c | 340 | 405 | 317 | 241 | 302 | 396 | 243 | 999 | 495 |
| 12 | 340 | 405 | 317 | 241 | 302 | 396 | 233 | 695 | 418 |
| 14a | 340 | 405 | 317 | 241 | 305 | 441 | 243 | 713 | 319 |
| 14b | 340 | 405 | 317 | 241 | 305 | 441 | 243 | 713 | 385 |
| 16 | 340 | 405 | 317 | 241 | 302 | 441 | 243 | 999 | 319 |
| 16a | 340 | 405 | 317 | 241 | 302 | 441 | 243 | 999 | 308 |
| 17a | 340 | 405 | 317 | 241 | 305 | 441 | 248 | 713 | 341 |
| 17b | 340 | 405 | 317 | 241 | 305 | **441** | 248 | 713 | 319 |
| 18 | 340 | 405 | 317 | 241 | 305 | 441 | 243 | 999 | 286 |
| 19 | 340 | 405 | 317 | 241 | 308 | 441 | 243 | 999 | 319 |
| 20 | 340 | 405 | 317 | 241 | 308 | 441 | 243 | 713 | 319 |
| 21 | 340 | 405 | 317 | 241 | 302 | 441 | 248 | 713 | 319 |
| 22 | 340 | 405 | 317 | 241 | 305 | 671 | 243 | 999 | 484 |
| 23b | 340 | 405 | 317 | 241 | 305 | 671 | 243 | 695 | 484 |
| 23c | 340 | 405 | 317 | 241 | 305 | 671 | 243 | 695 | 522 |
| 23d | 340 | 405 | 317 | 241 | 305 | 671 | 243 | 695 | 462 |
| 24a | 340 | 405 | 317 | 241 | 305 | **681** | 243 | 695 | 451 |
| 24b | 340 | 405 | 317 | 241 | 305 | 681 | 243 | 695 | 484 |
| 26a | 340 | 405 | 317 | 241 | 305 | 666 | 243 | 695 | 484 |
| 26b | 340 | 405 | 317 | 241 | 305 | 666 | 243 | 695 | 429 |
| 28a | 340 | 405 | 317 | 241 | 308 | 520 | 248 | 713 | 385 |
| 28b | 340 | 405 | 317 | 241 | 308 | 520 | 248 | 713 | 341 |
| 28c | 340 | 405 | 317 | 241 | 308 | 520 | 248 | 713 | **396** |
| 28d | 340 | 405 | 317 | 241 | 308 | 520 | 248 | 713 | 374 |
| 28e | 340 | 405 | 317 | 241 | 308 | 520 | 248 | 713 | 418 |
| 28f | 340 | 405 | 317 | 241 | 308 | 520 | 248 | 713 | 363 |
| 28g | 340 | 405 | 317 | 241 | 308 | 520 | 248 | 713 | **407** |
| 29 | 340 | 405 | 317 | 241 | 308 | 520 | 248 | 632 | 385 |
| 30a | 340 | 405 | 317 | 241 | 308 | 520 | 248 | 999 | 374 |
| 30b | 340 | 405 | 317 | 241 | 308 | 520 | 248 | 999 | 352 |
| 30c | 340 | 405 | 317 | 241 | 308 | 520 | 248 | 999 | 385 |
| 31 | 340 | 405 | 317 | 241 | 308 | 520 | 248 | 725 | 363 |
| 32a | 340 | 405 | 299 | 241 | 308 | 520 | 248 | 713 | 327 |
| 32b | 340 | 405 | 299 | 241 | 308 | 520 | 248 | 713 | 338 |
| 34a | 340 | 405 | 317 | 241 | 308 | 520 | 243 | 713 | 352 |
| 34b | 340 | 405 | 317 | 241 | 308 | 520 | 243 | 713 | 440 |
| 34c | 340 | 405 | 317 | 241 | **308** | 520 | 243 | 713 | 385 |
| 42a | 340 | 405 | 317 | 241 | 311 | 520 | 248 | 713 | 308 |
| 42b | 340 | 405 | 317 | 241 | 311 | 520 | 248 | 713 | 385 |
| 44b | 340 | 405 | 317 | **241** | 302 | 520 | 248 | 713 | 418 |
| 44c | 340 | 405 | 317 | **241** | 302 | 520 | 248 | 713 | 374 |
| 44d | 340 | 405 | 317 | 241 | 302 | 520 | 248 | 713 | 352 |
| 45 | 340 | 405 | 317 | 241 | 305 | 520 | 243 | 683 | 462 |
| 46a | 340 | 405 | **317** | 241 | 305 | 520 | 248 | 683 | 407 |
| 46b | 340 | 405 | 317 | 241 | 305 | 520 | 248 | 683 | 385 |
| 46c | 340 | 405 | 317 | 241 | 305 | 520 | 248 | 683 | 418 |
| 47a | 340 | 405 | 317 | 241 | 305 | 520 | 243 | 999 | **440** |
| 47c | 340 | 405 | 317 | 241 | 305 | 520 | 243 | 999 | 396 |
| 47d | 340 | 405 | 317 | 241 | 305 | 520 | 243 | 999 | 297 |
| 47g | 340 | 405 | 317 | 241 | **305** | 520 | 243 | 999 | 407 |
| 47h | 340 | 405 | 317 | 241 | 305 | 520 | 243 | 999 | **445** |
| 47i | 340 | 405 | 317 | 241 | 305 | 520 | 243 | 999 | **352** |
| 47j | 340 | 405 | 317 | 241 | 305 | 520 | 243 | 999 | **319** |
| 48a | 340 | 405 | 317 | 241 | 305 | 520 | 243 | 713 | 407 |
| 48b | 340 | 405 | 317 | 241 | 305 | 520 | 243 | 713 | 484 |
| 48c | 340 | 405 | 317 | 241 | 305 | 520 | 243 | 713 | 363 |
| 48d | 340 | 405 | 317 | 241 | 305 | 520 | 243 | 713 | 440 |
| 48e | 340 | 405 | 317 | 241 | 305 | 520 | 243 | 713 | 385 |
| 48f | 340 | 405 | 317 | 241 | 305 | 520 | 243 | 713 | 352 |
| 48g | 340 | 405 | 317 | 241 | 305 | 520 | 243 | 713 | 462 |
| 48h | 340 | 405 | 317 | 241 | 305 | 520 | 243 | 713 | 418 |
| 48i | 340 | 405 | 317 | 241 | 305 | 520 | 243 | 713 | 297 |
| 49a | 340 | 405 | 317 | **241** | 305 | 520 | 248 | 713 | **396** |
| 49b | 340 | 405 | 317 | 241 | 305 | 520 | 248 | 713 | 440 |
| 49c | 340 | 405 | 317 | 241 | 305 | 520 | 248 | 713 | 407 |
| 49d | 340 | 405 | 317 | 241 | 305 | 520 | 248 | 713 | 385 |
| 49e | 340 | 405 | 317 | 241 | 305 | 520 | 248 | **713** | 418 |
| 49f | 340 | 405 | 317 | 241 | 305 | 520 | 248 | 713 | 429 |
| 49g | 340 | 405 | 317 | 241 | 305 | 520 | 248 | 713 | 363 |
| 49h | 340 | 405 | 317 | 241 | 305 | 520 | 248 | 713 | 414 |
| 49k | 340 | 405 | 317 | 241 | 305 | 520 | 248 | 713 | **451** |
| 49p | 340 | 405 | 317 | 241 | 305 | 520 | 248 | 713 | 352 |
| 49q | 340 | 405 | 317 | 241 | 305 | 520 | 248 | 713 | 319 |
| 53 | 340 | 405 | **317** | 241 | 305 | 520 | 248 | 689 | 418 |
| 54 | 340 | 405 | 299 | 241 | 305 | 520 | 248 | 713 | 385 |
| 55a | 340 | 405 | 317 | 241 | 305 | 520 | 248 | 999 | 396 |
| 55c | 340 | 405 | 317 | 241 | 305 | 520 | 248 | 999 | 418 |
| 55d | 340 | 405 | 317 | 241 | 305 | 520 | 248 | 999 | 407 |
| 55e | 340 | 405 | 317 | 241 | 305 | 520 | 248 | 999 | 385 |
| 55f | 340 | 405 | 317 | 241 | 305 | 520 | 248 | 999 | 319 |
| 62a | 340 | 405 | 317 | 241 | **302** | 520 | 248 | 999 | 385 |
| 62b | 340 | 405 | 317 | 241 | 302 | 520 | 248 | 999 | 418 |
| 63 | 340 | 405 | 317 | 221 | 305 | 520 | 248 | 713 | 396 |
| 64 | 340 | 405 | 317 | 241 | 314 | 520 | 248 | 713 | 440 |
| 65 | 340 | 405 | 317 | 241 | 281 | 520 | 243 | 713 | 429 |
| 66 | 340 | 405 | 317 | 241 | 299 | 520 | 248 | 999 | 418 |
| 67a | 340 | 405 | 317 | 241 | 305 | 505 | 248 | 713 | 440 |
| 67b | 340 | 405 | 317 | 241 | 305 | 505 | 248 | 713 | 385 |
| 69 | 340 | 405 | 317 | 241 | 305 | 505 | 248 | 604 | **440** |
| 70 | 340 | 405 | 317 | 241 | 305 | 505 | 243 | 713 | 440 |
| 70a | 340 | 405 | 317 | 241 | 305 | 505 | 243 | 713 | 418 |
| 70b | 340 | 405 | 317 | 241 | 305 | 505 | 243 | 713 | 396 |
| 70c | 340 | 405 | 317 | 241 | 305 | 505 | 243 | 713 | 385 |
| 70d | 340 | 405 | 317 | 241 | 305 | 505 | 243 | 713 | 352 |
| 70e | 340 | 405 | 317 | 241 | 305 | 505 | 243 | 713 | 330 |
| 71 | 340 | 405 | 317 | 241 | 308 | 505 | 253 | 713 | 440 |
| 72 | 340 | 405 | 317 | 241 | 308 | 505 | 243 | 713 | 440 |
| 75 | 340 | 405 | 317 | 241 | 305 | 336 | 243 | 695 | 484 |
| 76 | 340 | 405 | 317 | 241 | 305 | 381 | 243 | 713 | 385 |
| 77 | 340 | 405 | 317 | 241 | 305 | 351 | 248 | 683 | 385 |
| 78 | 340 | 405 | 317 | 241 | 302 | 501 | 243 | 713 | 484 |
| 79 | 340 | 405 | 317 | 241 | 305 | 501 | 243 | 713 | 440 |
| 80 | **346** | 398 | 389 | 271 | 302 | 351 | 243 | 713 | 440 |
| 83 | 346 | 398 | 389 | 271 | 308 | 351 | 243 | 713 | 407 |
| 85 | 346 | 398 | 389 | 271 | 305 | 351 | 243 | 999 | 396 |
| 87 | 346 | 398 | 335 | 261 | 305 | 351 | 243 | 713 | 352 |
| 91 | 335 | 405 | 317 | 241 | 305 | 396 | 243 | 695 | 407 |
| 92 | 335 | 405 | 317 | 241 | 305 | 520 | 243 | 365 | 385 |
| 93 | 335 | 405 | 317 | 241 | 305 | 520 | 248 | 683 | 407 |
| 98 | 335 | 405 | 317 | 241 | 305 | 520 | 243 | 713 | 440 |
| 101 | 386 | 386 | 208 | 241 | 260 | 531 | 228 | 707 | 275 |
| 104 | 386 | 386 | 208 | 241 | 296 | 531 | 228 | 707 | 275 |
| 106a | 340 | 405 | 299 | 241 | 308 | 520 | 248 | 713 | 341 |
| 106b | 340 | 405 | 299 | 241 | 308 | 520 | 248 | 713 | 352 |
| 106c | 340 | 405 | 299 | 241 | 308 | 520 | 248 | 713 | 396 |
| 106d | 340 | 405 | 299 | 241 | 308 | 520 | 248 | 713 | 385 |
| 109 | 340 | 405 | 317 | 241 | 302 | 396 | 243 | 713 | 473 |
| 110 | 340 | 405 | 317 | 241 | 299 | 396 | 243 | 695 | 418 |
| 111a | 340 | 405 | 317 | 241 | 305 | 411 | 243 | 275 | **242** |
| 111b | 340 | 405 | 317 | 241 | 305 | 411 | 243 | 275 | 418 |
| 112 | 340 | 405 | 317 | 241 | 305 | 396 | 218 | 695 | 542 |
| 113 | 340 | 405 | 317 | 241 | 305 | 396 | 208 | 695 | 418 |
| 114 | 340 | 405 | 317 | 191 | 305 | 396 | 243 | 695 | 385 |
| 115 | 329 | 405 | 317 | 241 | 305 | 396 | 238 | 695 | 418 |
| 116 | 340 | 405 | 317 | 241 | 299 | 671 | 243 | 999 | 484 |
| 117 | 340 | 405 | 317 | 241 | 305 | **486** | 243 | 695 | 451 |
| 118 | 340 | 405 | 347 | 241 | 305 | 520 | 248 | 713 | 418 |
| 119 | 340 | 405 | 317 | 245 | 305 | 520 | 248 | 713 | 418 |
| 121 | 340 | 405 | 317 | 171 | 305 | 520 | 248 | 713 | 363 |
| 122 | 340 | 405 | **303** | 241 | 305 | 520 | 248 | 713 | 418 |
| 123 | 340 | 405 | 317 | 241 | 305 | 396 | 243 | 689 | 385 |
| 124 | 340 | 405 | 317 | 241 | 305 | 396 | 243 | 683 | 385 |
| 125 | 340 | 405 | 317 | 241 | 305 | 396 | 248 | 695 | 418 |
| 126 | 340 | 405 | 317 | 241 | 302 | 441 | 243 | 713 | 319 |
| 127 | 340 | 405 | 317 | 241 | 308 | 520 | 243 | 683 | 440 |
| 128 | 340 | 405 | **341** | 241 | 308 | 520 | 248 | 683 | 385 |
| 129 | 340 | 405 | **299** | 241 | 308 | 520 | 248 | 683 | 385 |
| 130 | 340 | 405 | 341 | 241 | 308 | 520 | 248 | 713 | 352 |
| 131 | 340 | 405 | 341 | 241 | 308 | 520 | 248 | 701 | 363 |
| 132 | 340 | 405 | 317 | 241 | 308 | 520 | 248 | 701 | 363 |
| 133 | 340 | 405 | 317 | 241 | 311 | 520 | 248 | 999 | 385 |
| 134 | 340 | 405 | 317 | 241 | 305 | 520 | 248 | 725 | 440 |
| 135 | 340 | 405 | 317 | 241 | 305 | 520 | 248 | 638 | 440 |
| 136a | 340 | 405 | **317** | 241 | 305 | 520 | 248 | 653 | **440** |
| 136b | 340 | 405 | **317** | 241 | 305 | 520 | 248 | 653 | 330 |
| 137 | 340 | 405 | 317 | 241 | 305 | 520 | 280 | 999 | 418 |
| 138 | 340 | 405 | **317** | 241 | 305 | 520 | **258** | 713 | 440 |
| 139 | 340 | 405 | **341** | 241 | 305 | 520 | 248 | 683 | 418 |
| 140 | 340 | 405 | 317 | 241 | 305 | 520 | 258 | 647 | 440 |
| 141 | 340 | 405 | 341 | 241 | 305 | 520 | 258 | 647 | 440 |
| 142 | 340 | 405 | 317 | 241 | 305 | 505 | 243 | **689** | 440 |
| 143 | 340 | 405 | 317 | 241 | 305 | 505 | 243 | **683** | 440 |
| 144 | 340 | 405 | 317 | 241 | 302 | 681 | 243 | 695 | 484 |
| 145 | 340 | 405 | 317 | 241 | 302 | 666 | 243 | 695 | 484 |
| 146 | 346 | 398 | 389 | 271 | **302** | 351 | 243 | 999 | 407 |
| 147 | 346 | 398 | 389 | 271 | **293** | 351 | 243 | 999 | 407 |
| 148 | 346 | 398 | 389 | 261 | 302 | 351 | 243 | 713 | 407 |
| 149 | 346 | 398 | 389 | 261 | 305 | 351 | 243 | 713 | 407 |
| 150 | 346 | 398 | 335 | 261 | 302 | 351 | 243 | 713 | 352 |
| 151 | 335 | 405 | 317 | 241 | 305 | 396 | 243 | 683 | 407 |
| 152 | 335 | 405 | 317 | 241 | 305 | 396 | 243 | 689 | 407 |
| 153 | 335 | 405 | 317 | 241 | 308 | 520 | 248 | 713 | 352 |
| 154 | 335 | 405 | 317 | 241 | 308 | 520 | 248 | 683 | 352 |
| 155 | 335 | 398 | 317 | 241 | 305 | 520 | 248 | 713 | 440 |
| 156 | 335 | 398 | 317 | 241 | 305 | 520 | 248 | 683 | 440 |
| 157a | 317 | 386 | 208 | 191 | 299 | 531 | 228 | 707 | **363** |
| 157b | 317 | 386 | 208 | 191 | 299 | 531 | 228 | 707 | **374** |
| 159a | 335 | 405 | 317 | 241 | 305 | 520 | 243 | 999 | 440 |
| 159b | 335 | 405 | 317 | 241 | 305 | 520 | 243 | 999 | **407** |
| 160 | 386 | 386 | 208 | 241 | 299 | 531 | 243 | 707 | 275 |
